# Doubled Haploid ‘CUDH2107’ as a Reference for Bulb Onion (*Allium cepa* L.) Research: Development of a Transcriptome Catalogue and Identification of Transcripts Associated with Male Fertility

**DOI:** 10.1371/journal.pone.0166568

**Published:** 2016-11-18

**Authors:** Jiffinvir S. Khosa, Robyn Lee, Sophia Bräuning, Janice Lord, Meeghan Pither-Joyce, John McCallum, Richard C. Macknight

**Affiliations:** 1 Department of Biochemistry, University of Otago, Dunedin, New Zealand; 2 Department of Botany, University of Otago, Dunedin, New Zealand; 3 New Zealand Institute for Plant & Food Research, Lincoln, New Zealand; Youngstown State University, UNITED STATES

## Abstract

Researchers working on model plants have derived great benefit from developing genomic and genetic resources using ‘reference’ genotypes. Onion has a large and highly heterozygous genome making the sharing of germplasm and analysis of sequencing data complicated. To simplify the discovery and analysis of genes underlying important onion traits, we are promoting the use of the homozygous double haploid line ‘CUDH2107’ by the onion research community. In the present investigation, we performed transcriptome sequencing on vegetative and reproductive tissues of CUDH2107 to develop a multi-organ reference transcriptome catalogue. A total of 396 million 100 base pair paired reads was assembled using the Trinity pipeline, resulting in 271,665 transcript contigs. This dataset was analysed for gene ontology and transcripts were classified on the basis of putative biological processes, molecular function and cellular localization. Significant differences were observed in transcript expression profiles between different tissues. To demonstrate the utility of our CUDH2107 transcriptome catalogue for understanding the genetic and molecular basis of various traits, we identified orthologues of rice genes involved in male fertility and flower development. These genes provide an excellent starting point for studying the molecular regulation, and the engineering of reproductive traits.

## Introduction

Bulb onion (*Allium cepa* L.) is a monocot vegetable crop grown for edible bulbs and has economic importance worldwide. The onion research community would benefit from improved onion genomic resources [1, 2]. In recent years, next generation sequencing technologies have been used in crop plants to generate genomic and transcriptomic data sets in a cost and time-effective manner. The use of RNA sequencing (RNA-seq) enables researchers to discover genes and molecular markers associated with important traits for their breeding programmes [3, 4]. RNA-seq is a particularly attractive approach in non-model crops that have large genomes, where genomic sequencing is complex and expensive. To aid downstream analysis and avoid detection of false SNPs, a good quality transcriptome assembly is essential [3, 5, 6]. However in species that are highly heterozygous, such as bulb onion, the development of a high quality reference transcriptome is challenging, as it is hard to distinguish the transcripts belonging to different members of a gene family from allelic variants of a particular gene [2, 3, 5]. In out-crossing crop plants, such as bulb onion, the complications of heterozygosity can be overcome by using homozygous double haploids [1, 6]. Double haploid (DH) lines have been developed in bulb onion and have proved useful for various genetic and genomic studies [7–10]. There are many advantages in the use of a common reference double haploid line for genetic and genomic studies by researchers throughout the world. Unfortunately the majority of onion DH lines are neither vigorous nor have good seed production, which complicates wider distribution and usage. In contrast, a set of DH lines developed from a synthetic background at Cornell University [7] have proved to be more widely usable for breeding [11]. We have suggested that ‘CUDH2107’ be employed as a common reference line, as it is relatively vigorous, produces adequate amounts of seed and produces a bulb that stores well [12].

The development of F_1_ hybrid onions has transformed the quality and yield of onion production. However, there are concerns that the introduction of F_1_ hybrids has reduced the diversity of germplasm being grown. As only two sources of male sterility (CMS-S and CMS-T) have been utilized, it would be desirable to identify additional sources of male sterility in bulb onion [2]. In other plant species, wide hybridization and induced mutagenesis have been utilized to develop male sterile phenotypes [13]. The male sterile mutants often have either abnormal development of sporophytic anther tissues (primarily tapta and meiotic cells) causing lack of pollen or pollen abortion, or have abnormal development of gametophytic anther tissues affecting microspore or pollen grain formation. There is a large body of research into the genetics and molecular mechanisms of male sterility and fertility restoration in other plants, especially monocots, such as rice and maize that could potentially be applied to onion [13–15]. Recently, the CMS-S onion mitochondrial genome was sequenced, leading to the finding that orf725 might be the most plausible candidate gene responsible for inducing male sterility [16]. Further, a gene encoding PMS1, involved in the DNA mismatch repair pathway, was identified as the possible candidate gene regulating fertility restoration [17]. However, the molecular mechanisms of male sterility and fertility restoration in bulb onion is still poorly understood [2].

In this paper, we develop a transcriptome catalogue for ‘CUDH2107’ as a resource for the *Allium* research community [12]. To demonstrate the utility of this data, we identified orthologues of rice genes involved in male fertility and restoration of CMS, which could be useful for studying these processes in bulb onion. This information provides potential targets for the development of novel sources of male sterility for hybrid seed production, by using new genome editing such as CRISPR/cas9 to induce specific mutations in these genes.

## Material and Methods

### Plant material and transcriptome sequencing

Seed lots of the long day DH bulb onion ‘CUDH2107’ (line CUDH066607 in [11]) were provided by Cornell University (US). Total RNA was extracted from tissue samples pooled from multiple plants grown in tunnel houses at Lincoln New Zealand (latitude 42° S) or in controlled environments. The stages sampled were as follows: leaves (from plants grown in long day of 16 h light: 8 h dark), floral buds from unexpanded umbels, unopened florets from expanded umbels, open florets with pollen, older flowers and roots. RNA was isolated using a Qiagen RNA extraction kit following the manufacturer’s guidelines. Libraries were made using the TruSeq v2 kit (Illumina), and were sequenced on the Illumina HiSeq 2000 platform by NZGL Ltd.

### *De novo* assembly of transcripts

The program fastq_quality_trimmer (FASTX_toolkit, version 0.0.13) was used to trim bases with a quality score less than 30, subsequently any reads containing shorter than 20 bases were removed. The cleaned reads from the CUDH2107 onion tissue libraries were assembled together in a single reference *de novo* assembly using Trinity [18] following the protocol and default parameters [19]. The combined *de novo* assembly is referred to as the ‘extensive transcriptome dataset’, which was filtered based on a minimum fragments per kb of target transcript length per million (FPKM) value of 0.5 (41 reads per kb). As a result we compiled the ‘abundant transcriptome dataset’ of highly expressed transcripts, which was used in all the analyses described in this paper. All the sequence data is deposited at NCBI as sequence read archive (S1 Table).

The completeness of the extensive and abundant transcriptomes was assessed based on assembly statistics achieved by running the script ‘TrinityStats.pl’ [18]. In addition, the eukaryotic Benchmarking Universal Single-copy Orthologs (BUSCOs) dataset (http://busco.ezlab.org/, accessed on 20 May 2015) was compared with our abundant transcriptome dataset using BUSCO_v1.1 [20].

### Sequence conservation and functional annotation

Standalone BLAST (ncbi-blast-2.2.27+, [21] was used to perform sequence similarity searches of the current onion transcriptome assembly to a variety of transcriptome assemblies and rice proteins. BLASTN with an E-value cut off of 10^−4^ was used to estimate the sequence conservation among rice and other transcriptomic assemblies of onion, bunching onion, and garlic [8, 10, 22–25]. BLASTX search with an E-value cut off of 10^−4^ was used to compare the peptides encoded by the onion transcripts to rice proteins [26] and the results were used to obtain Gene Ontology (GO) terms for the onion transcripts. This was achieved using GO annotations identifiers from the Rice Genome Annotation Project (http://rice.plantbiology.msu.edu/downloads_gad.shtml).

To further identify transcripts potentially coding for full-length peptides, the abundant transcriptome dataset was screened for Open Reading Frames (ORFs) using the ORF-predictor server [27] http://proteomics.ysu.edu/tools/OrfPredictor.html). The resulting predicted peptides were filtered, using custom python and R scripts (available on request), to only retain transcripts with predicted peptides that are at least 100 amino acids long.

### Abundance estimation and differential expression analysis

The trinity protocol was followed for abundance estimation, differential expression and hierarchical clustering [19]. Transcript abundance was calculated by first aligning the trimmed reads from each sample to the extensive transcriptome dataset using Bowtie then RSEM [28] was used to estimate abundance of each transcript. The differential transcript expression between different samples was calculated using the Bioconductor package EdgeR [29]. To compare transcriptional profiles across samples, transcripts differentially expressed in at least one pairwise comparison were used to perform hierarchical clustering of transcripts and samples. For the hierarchical clustering, the FPKM values (obtained from RSEM) were log_2_-transformed and median-centered. To compare correlation between each sample pair, TMM (Trimmed Mean of M-values) normalized FPKM values were used to obtain a Spearman correlation matrix, then the correlation matrix was hierarchically clustered and visualized as a heat map.

### Identification of male fertility genes

The coding DNA sequence of rice flowering genes [26] was used as query to perform BLASTn against bulb onion transcriptome data with an E value cut off of 1e^-4^. Top blast hits from bulb onion were translated and use as query sequence in reciprocal BLASTp searches against rice database. The bulb onion contigs retrieving rice genes after reciprocal blast were selected for further analysis. The multiple sequence alignment using amino acid sequences was carried out with GENEIOUS 6.1. The aligned sequences were used for generating trees based on Neighbour Joining Method in the GENEIOUS 6.1 software package. The relative expression of bulb onion genes in different samples was calculated based on FPKM values.

### Quantitative real-time PCR

The differential expression of genes involved in flower development was validated using qPCR. Total RNA was isolated from different development stages using Plant RNA Purification Reagent (Invitrogen, USA) following manufacturer’s guidelines. Reverse transcription was carried out with 1µg of total RNA using Invitrogen Super Script III following manufacturer’s guidelines. Quantitative real time PCR was carried out using 10µL SYBR reaction mixture (Kapa Biosystems) in a Roche Light Cycler 480. Relative gene expression levels were calculated using the 2 (2 delta delta C (T) method in Roche LC480 software. Actin and ß-tubulin were used as the reference genes. The list of primer sequences used in present investigation was given in S2 Table.

## Results and Discussion

### Transcriptome of bulb onion and its comparison with other alliums

**Fig 1:**
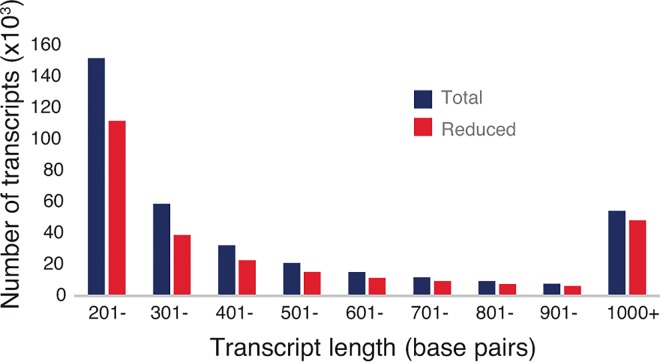
Length distribution of assembled transcripts in the extensive (Total) and abundant (Reduced) transcriptome datasets.

Illumina sequencing was carried out on cDNA libraries developed from leaves, immature flower heads, unopened flowers, opened flowers with pollen, older flowers, and roots. This resulted in approximately 396 million 100 base pair paired reads. Using Trinity software, cleaned reads were *de novo* assembly into 362,106 contigs representing what we called the ‘extensive transcriptome dataset’ of bulb onion. Transcripts had a total length of 218.6 Mbp with an average length of 603bp and N50 length of 901bp. We filtered out the low abundance contigs and mostly short contigs from the extensive transcriptome dataset using a minimum FPKM value of 0.5, which represents an average base coverage of 8.2. This resulted in 271,665 highly expressed contigs with an average length of 653bp and N50 length of 1055bp (combined transcript length 177Mbp) (Fig 1; Table 1). In the abundant transcriptome dataset, 266,427 transcripts were predicted to encode peptides, with 50,220 transcripts encoding peptides that are at least 100 amino acids long.

**Table 1:** Statistics of De novo assembly and abundance estimation.

	Extensive (Total)	Abundant (Reduced)
Number of Transcripts	362,106	271,665
Total transcripts length (bp)	218,643,405	177,424,188
Average Transcript length (bp)	603.81	653.10
Median Transcript length (bp)	341	355
Minimum Transcript length (bp)	201	201
Maximum Transcript Length (bp)	20,231	20,231
N50 (bp)	901	1055
GC %	36.96	37.12

Only 15% to 49% of the highly abundant transcripts identified by this study were similar to those previously identified in bulb onion [8–10, 23], highlighting the value of our multiple organ transcriptome assembly. The half of the transcripts from this transcriptome assembly were present in an assembly from six week old seedling short and long day onions [23]. Also in garlic, 78% of transcripts generated in multiple organ transcriptome were present in the transcriptome developed from single tissue [22, 24]. Transcriptome assemblies have been developed from other economically important *Allium* species (bunching onion and garlic) and their comparison with bulb onion gives us an idea about the degree of transcriptome conservation in the genus *Allium* [22, 24, 25]. Only 22% and 10% of bulb onion transcripts were highly similar to transcripts from bunching onion and garlic transcriptomes, respectively. However, those that were similar shared 93% identity with bunching onion and 90% with garlic (Table 2), supporting the fact that bulb onion is more closely related to bunching onion than to garlic [30]. The common transcripts identified between different alliums in the present investigation will be useful for better understanding of *Allium* comparative genomics.

**Table 2:** Sequence conservation between the bulb onion abundant transcriptome dataset and other *Allium* species.

Species	Reference database^a^	Number of Reference sequences	Number and % of hits in reference database	Average Identity (%) of hits	Reference
Bunching onion	274623	54,903	59,923 (22.06)	93.63	25
Bulb onion	246669	367,683	133,547 (49.16)	98.04	23
Bulb onion	238142	128,598	114,151 (42.02)	98.79	8
Bulb onion	175446	33,162	52,297 (19.25)	97.59	10
Bulb onion	175449	26,995	42,264 (15.56)	97.58	10
Garlic	158177	79,143	27,383 (10.08)	90.92	22

^a^ http://www.ncbi.nlm.nih.gov/bioproject/

### Gene prediction and functional annotation

A total of 56,805 (20.91%) transcripts showed significant hits with rice proteins and shared 56% average identity. To further validate the gene predictions, we used the predicted bulb onion peptides to search KOGs, the core genes from the Benchmarking Universal Single-Copy Orthologues (BUSCOS) pipeline [20]. This search revealed that the transcriptome contains 82% complete BUSCOs (250) and 4% fragmented BUSCOs and indicates a near complete transcriptome.

**Fig 2:**
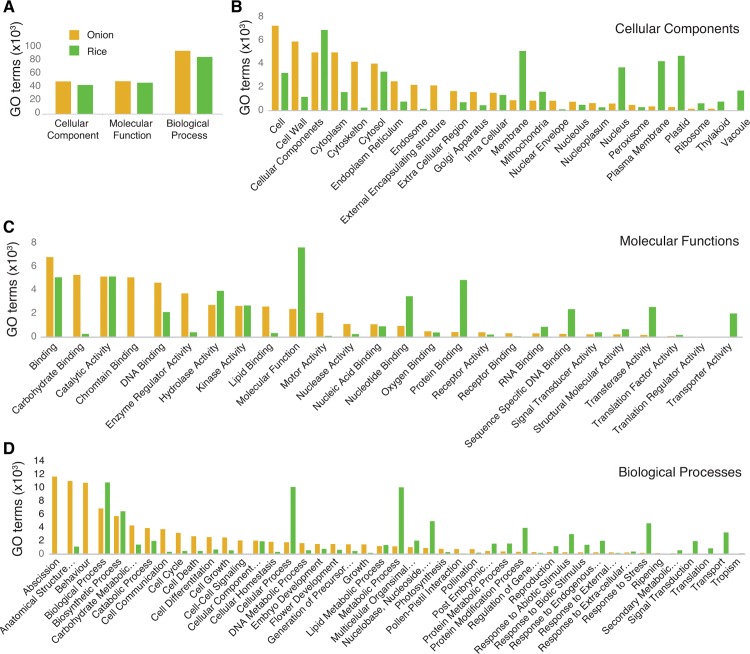
GO terms in bulb onion compared with rice. (A) Total number of GO terms associated with cellular component, molecular function and biological process in onion (brown) and rice (green). (B-D) GO terms in onion (brown) and rice (green) associated with; (B) cellular component, (C) molecular function, and (D) biological process.

All predicted bulb onion peptides were functionally annotated following a consensus approach using GO slims from the rice genome annotation database. The bulb onion transcripts were grouped into 95 functional groups (Cellular component, Biological Process and Molecular Function) A similar number of GO terms were found in bulb onion and rice but the number of genes with GO terms in various categories differed (Fig 2A), which might reflect differences in life cycle, development stages and physiological pathways [31]. We found 24 categories within ‘Cellular Components’, 26 within ‘Biological Process’ and 45 within ‘Molecular Function’ categories (Fig 2). The top GO terms for ‘Cellular Component’ were cell (7267), followed by cell wall (5912) and cellular components (4987) (Fig 2B). For ‘Biological Processes’, top GO terms were Abscission (11,710), followed by Anatomical Structure Morphogenesis (11,054) and Behaviour (10,775) (Fig 2C). In the case of ‘Molecular Function’, Binding Domains (6811) was the most abundant GO term, followed by Carbohydrate Binding (5294) and Catalytic Activity (5152) (Fig 2D).

### GC content

GC content is a striking characteristic of genome organization and life history of plant species [32]. Bulb onion has a lower GC content than grasses, which might be due to the large genome size found in bulbous geophytes [32, 33]. The average GC content in the present transcriptome dataset is ~38%, whereas bunching onion transcriptome have 40% GC content [25]. The GC content in present investigation is lower than that previously reported based on small EST dataset [33]. This difference might be due to variation in gene length, structure, expression and methylation in these datasets, as these factors affect GC content [34]. Overall our findings confirm the occurrence of low GC content in genus *Allium*.

### Tissue specific expression

**Fig 3:**
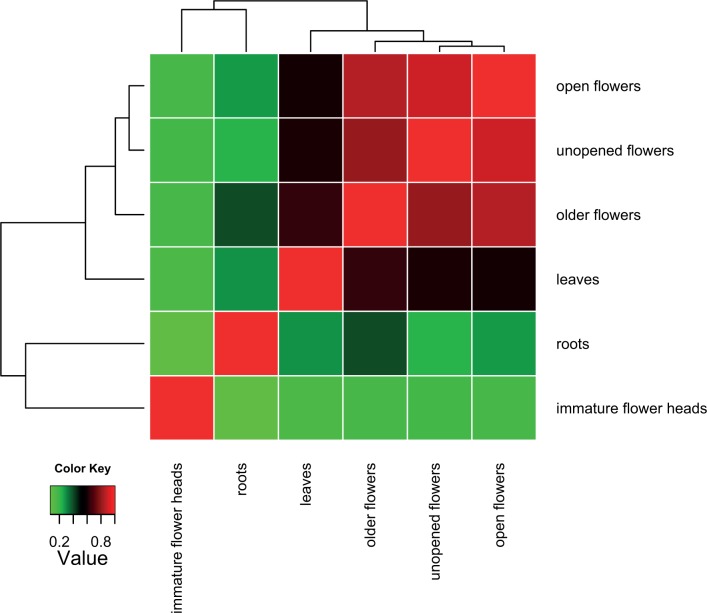
Comparisons of transcriptional profiles across samples. Heat map showing hierarchical clustered Spearman correlation matrix resulting from a pairwise comparison of transcript expression values.

To study the expression pattern of transcripts across different bulb onion tissues, pairwise comparisons were used to identify transcripts that are differentially expressed in at least one tissue. Using a significance threshold of 0.001 False Discovery Rate and 4-fold change in expression, we determined that there were 17 thousand transcripts differentially expressed among different tissues. ‘Unopened flowers’ and ‘open flowers with pollen’ shared a more similar pattern of expression, with the next most similar sample being ‘older flowers’ (Fig 3). However these samples demonstrated a quite different pattern of expression to that of ‘immature flower heads’ (Fig 3). Transcripts from leaves and roots also showed distinct expression patterns, as they grouped on separate nodes (Fig 3).

### Transcription factors in bulb onion

**Fig 4:**
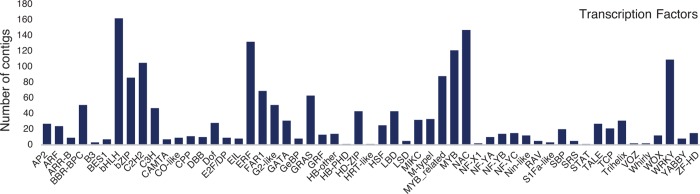
Number of onion sequence contigs encoding transcription factors belonging to different families.

Transcription factors play an important role in plant development and stress responses [35]. A wide range of TFs have been identified and characterized in different plant species [36]. We identified 1837 bulb onion transcripts encoding orthologues of rice transcription factors and grouped into 55 families (Fig 4). The most highly represented transcription factor families were bHLH (162 transcripts), NAC (147 transcripts), EFR (132 transcripts), MYB (121 transcripts), WRKY (109 transcripts) and C2H2 (105 transcripts) (Fig 4). These transcription factors regulate various processes of flower development; functional characterization of these genes will allow us to have a better understanding of bulb onion growth and development to enhance onion breeding programmes [35].

### Identification and expression analysis of male fertility genes

Male reproductive development and fertility are important agronomical traits in crop plants. The identification of genes involved in these processes allows better understanding of the molecular mechanism of male fertility [13] and will assist breeders to develop male sterile lines to utilize in heterosis breeding [13, 37]. Using our transcriptome data which was derived from developing flower buds and flowers (and other tissues) of normal male fertile plants, we found potential orthologues of a range of rice genes involved in male fertility and flower development (Table 3). These genes are also present in other plant species and have conserved functions indicating common mechanisms of flower development [38–39].

**Table 3:** Bulb onion orthologues of rice genes involved in male fertility and floral development identified using BLAST searches.

Gene Name	Bulb onion contigs^a^	Rice	% a.a Identity	Mutant	References
*CARBON SATRVED ANTHERS* (*CSA*)	c244438_g1_i1	LOC_Os01g16810	51.70	Reduced levels of carbohydrates in anthers and it causes male sterility.	[40]
*PISTILLATA* (*OsMADS4*)	c95167_g1_i1	LOC_Os5g34940	55.50	Abnormal lodicule development	[41]
*AGAMOUS (OsMADS3)*	c34046_g1_i1	LOC_Os01g10504	60.40	Defective anther wall, aborted microspores	[42]
*SEPALLATA3 (OsMADS7)*	c216523_g1_i1	LOC_Os08g41950	53.50	homeotic changes of lodicules, stamens and carpels into palea/lemma-like organs, and a loss of floral determinacy.	[43]
*AGAMOUS LIKE 6 (OsMADS6)*	c129731_g1_i1	LOC_Os02g45770	70.90	Abnormal palea and lodicule development	[44]
*TGA9*	c151564_g1_i1	LOC_Os11g05480	66.40	Abnorlam anther	[45]
*JAGGED (STAMENLESS)*	c30443_g1_i1	LOC_Os01g03840	48.40	Abnormal lemma and palea, Defective stamen	[46]
*MPK3*	c121397_g1_i1	LOC_Os03g17700	82.90	Defective anther	[47]
*MPK6*	c123481_g1_i1	LOC_Os06g06090	86.70	Defective anther	[47]
*SWI1 (OsAM1)*	c201101_g1_i1	LOC_Os03g44760	50.00	Arrested Meiosis at earlyt prophase 1	[48]
*TPD1 (TDL1A)*	c21970_g1_i1	LOC_Os12g28750	47.20	Multiple megaspore mother cells	[49]
*EMS1 (MSP1)*	c101027_g1_i1	LOC_Os01g68870	69.70	Excessive number of male and female sporocytes, lack of the tapetum	[50]
*MMD1*	c3619_g1_i1	LOC_Os03g50780	42.10	Male sterile due to a defect in meiosis II	[51]
*WAX-DEFICIENT ANTHER 1*	c115430_g1_i1	LOC_Os10g33250	64.70	Defect in pollen exine formation	[52]
*REDUCED ADH ACTIVITY*	c78051_g1_i1	LOC_Os09g35000	50.70	-	-
*PERSISTENT TAPETAL CELL1*	c179910_g1_i1	LOC_Os09g27620	45.60	Mutants fail to make functional pollen; pollen degenerates after microspore release and the tapetum also appears abnormally vacuolated.	[53]
*AP3 SUPERWOMAN1* (*OsMADS16*)	c229753_g1_i1	LOC_Os06g49840	67.80	transformation of the lodicule and the stamen into mrp- and carpel-like organs, respectively	[54]
*ENT-KAURENE OXIDASE*	c117603_g1_i2	LOC_Os06g02019	57.80	Defects in exine formation	[55]
*RAS-RELATED NUCLEAR PROTEIN GTPASE*	c112324_g1_i1	LOC_Os05g49890	92.30	-	-
*IMPORTIN BETA1*	c223739_g1_i1	LOC_Os05g28510	69.60	-	-
*ATSIZ1/SIZ1*	c119191_g13_i1	LOC_Os05g03430	75	-	-
*SOLO DANCERS*	c104859_g1_i1	LOC_Os03g12414	25.20	Defects in homolog interaction, bivalanet and meiotic crossover formation	[56]
*SUCROSE TRANSPORTER*	c87072_g1_i1	LOC_Os03g07480	66.80	-	-
*CYTOCHROME P-450B*	c74282_g1_i1	LOC_Os03g07250	68.10	Aborted pollen grains	[57]
*MYOSIN XI B*	c106734_g1_i1	LOC_Os02g57190	68.40	male sterility under short-day-length (SD) and fertility under long-day-length (LD) conditions	[58]
*APOPTOSIS INHIBITOR 5*	c81284_g1_i1	LOC_Os02g20930	66.80	Inhibition of tapetal PCD and aborted pollen	[59]
*ENT-KAURENE SYNTHASE*	c83938_g1_i2	LOC_Os02g17780	58.60	-	-
*SHOOTLESS2*	c78229_g1_i2	LOC_Os01g34350	59.30	Abnormal stamen development	[60]
*DMC1*	c43784_g1_i1	LOC_Os11g04954	87.10	Defects synapsis and crossing over	[61]
*ZEP1*	c116971_g1_i3	LOC_Os04g37960	44.60	Defects in synaptoneal complex assembly	[62]
*POLLEN SEMI-STERLITY1*	c115973_g1_i1	LOC_Os08g02380	40	Reduced pollen viability and anther dehiscence	[63]
*REC8*	c107330_g1_i3	LOC_Os01g67250	26.80	Defects in homologous chromosome pairing and telomere formation	[64]
*LIS1*	c65717_g1_i1	LOC_Os08g06480	81.20	Defects in male gametophyte and male sterlity	[65]

^a^ The sequence of these contigs is provided in S3 Table.

**Fig 5:**
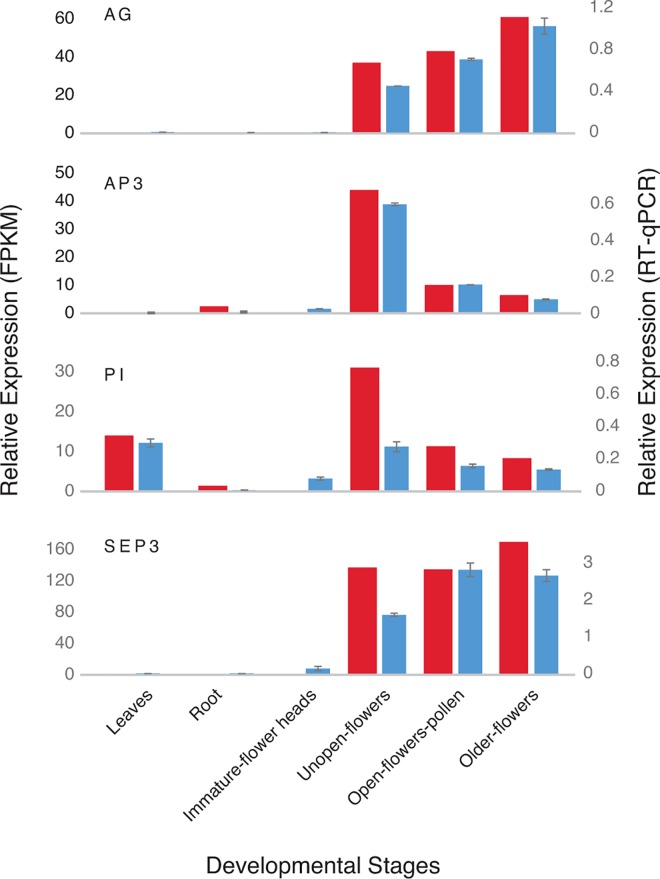
Relative expression of the floral meristem identity genes *AGAMOUS* (*AG*), *APETALLA3* (*AP3*), *PISTILLATA* (*PI*) and *SEPALLATA3* (*SEP3*) across different onion organs. Expression determined from the RNAseq data is shown in red and RT-PCR data is shown in blue, with data represented by an average ± S.E. of three samples, with transcripts normalized to actin and β-tubulin.

We identified a number of flower development genes, including the MADS box genes *PISTILLATA*, *AGAMOUS*, *SEPALLATA3*, *APETALLA3* and *AGAMOUS LIKE*6. These MADS box genes determined flower organ identity, and mutations in some of these genes can result in male sterility [66–69]. The expression pattern of floral meristem genes varies in different developmental stages across a wide range of plants [31, 70–71]. We found that *AGAMOUS*, *AGL6*, *AP3* and *SEPALLATA3* were expressed in bulb onion flowers (from unopened flowers to older fully open flowers) but not in immature flower heads (Fig 5). *PISTILLATA* had a similar expression pattern to *AP3* and the floral meristem identity genes, but was also detected at relatively high levels in bulb onion leaves (Fig 5). *PISTILLATA* has been found to be also expressed in the leaves and roots in different plants but their function in vegetative organs is still unknown [72–75]. The flower specific MADS genes we have identified in bulb onion could be mutated to generate male specific mutants. For example, the rice *AGAMOUS* (also known as *OsMADS3*) mutant plants show severe defects in stamen identity and lodicule number which leads to male sterility [76]. A naturally occurring mutation induced by retrotransposon insertion in *OsMADS3* has recently been identified, which causes recessive male-sterility but retains good agronomical performance, so it could be used as an elite line for recurrent selection [68].

The timely degradation of tapetal cells is a prerequisite for the development of viable pollen grains. *PERSISTANT TAPETAL CELL1* (*PTC1*) is a rice orthologue of Arabidopsis *MALE STERILITY1* (*MS1*) gene encoding a Plant Homeodomain (PHD) protein that regulates programmed tapetal development and pollen formation [77–79]. *MALE MEIOCYTE DEATH1* (*MMD1*) is another PHD protein involved in the regulation of gene expression during meiosis mutations [51]. Mutations in these genes results in complete male sterility in *Arabidopsis*, rice and barley [51, 77–80]. In the bulb onion transcriptome dataset we found two contigs having characteristic PHD domains encoding *PERSISTANT TAPETAL CELL1* (*PTC1*) and *MALE MEIOCYTE DEATH1* (*MMD1*). Other transcripts encoding proteins that are required for male fertility are listed in Table 3 (the sequences of onion contigs corresponding to these genes is given in S3 Table). As the molecular mechanism controlling floral development is largely conserved across plant species [38–39], some of the candidate male fertility genes we have identified in bulb onion could provide excellent targets for engineering new male sterile lines.

A new method of generating hybrid seed has been developed in maize [81]. This involves identifying or generating a male sterile mutant and then adding three transgenes to firstly complement the mutant to recover male fertile plants; second to prevent pollen formation so that the restoration of male ferility can only be maternally inherited, and thirdly to provide a easily detected fluorescent reporter protein ensuring any contaminating seed containing the transgenes is easily detected [81]. This system provides a simple way of generating male sterile female plants for hybrid seed production, however it is necessary to first generate a male sterile mutant. This has now been done using the CRISPR/Cas9 genome editing technique [82–83].

## Conclusions

High heterozygosity and inbreeding depression hampers onion improvement and genetic programs but can be counteracted by using double haploid lines in genetic and molecular biology research projects. In this context we developed a transcriptome dataset using double haploid “CUDH2107” as reference line to provide more genomic resources for the *Allium* research community. The development of a transcriptome assembly from different development stages of bulb onion is a valuable genomic resource for better understanding the genetic and molecular basis of various traits. In the present investigation, a transcriptome dataset has been generated from different vegetative and reproductive organs. This dataset was explored to identify genes involved in male fertility and examine their expression in different organs. The next step would be to functionally characterize these genes to identify those that could be mutated to develop male sterile lines for hybrid production. A variety of approaches have been used for the production of a transgenic male sterility–fertility restoration system [37, 81–86]. Targeted mutagenesis has been utilized in maize to induce mutations in male fertility genes [81–83]. The use of genome editing techniques, such as CRISPR/cas9, provides a new way to induce specific mutations in genes regulating anther and pollen development. The ability to engineer sterility in bulb onion would remove the limitation of using a single source of male sterility (CMS-S), and could broaden the genetic base of F_1_ hybrids.

## Supporting Information

S1 Table Description of samples used and their NCBI SRA links. (DOCX)

S2 Table List of primer Sequences used in present investigation. (DOCX)

S3 Table Sequences in FASTA of bulb onion orthologues of rice genes involved in male fertility and floral development identified using BLAST searches. (XLSX)
