# Supplementary material for: Doubled Haploid ‘CUDH2107’ as a Reference for Bulb Onion (Allium cepa L.) Research: Development of a Transcriptome Catalogue and Identification of Transcripts Associated with Male Fertility
Source: PLoS One. 2016 Nov 18;11(11):e0166568. doi: 10.1371/journal.pone.0166568 (PMC5115759; doi:10.1371/journal.pone.0166568)
Supplement: S1 Table — (DOCX) [file pone.0166568.s001.docx]

**Supporting Information 1**

**S1 Table 1. Description of samples used and their NCBI SRA links.**

| **Sample** | **Description** | **NCBI Biosample number ^a^** |
| --- | --- | --- |
| Leaves | Leaves from 3 month old plants grown under long days. Tissue was taken ~2 cm from leaf tip. | 5898004 |
| Roots | Whole roots from 3 month old plants | 5898005 |
| Immature flower heads | Small immature flower head from 14 month old plants - floral buds not visible without dissection. | 5898006 |
| Un-open Flowers | Flowers clearly formed but unopened from 14 month old plants | 5898007 |
| Open flowers with pollen | Opened flowers with pollen from 15 month old plants | 5898008 |
| Older flowers | Flowers that are all open from 15.5 month old plants | 5898009 |

**^a^ Retrieve at** www.ncbi.nlm.nih.gov/biosample
