# Supplementary material for: Doubled Haploid ‘CUDH2107’ as a Reference for Bulb Onion (Allium cepa L.) Research: Development of a Transcriptome Catalogue and Identification of Transcripts Associated with Male Fertility
Source: PLoS One. 2016 Nov 18;11(11):e0166568. doi: 10.1371/journal.pone.0166568 (PMC5115759; doi:10.1371/journal.pone.0166568)
Supplement: S2 Table — (DOCX) [file pone.0166568.s002.docx]

**Supporting Information 2**

**S2 Table 2. List of primer Sequences used in present investigation**

| Primer Name | Forward Sequence | Reverse Sequence |
| --- | --- | --- |
| AcAG | CGCTTGTAGATGCAGTTGGC | GCAGCTTGAAACCAGACTCG |
| AcPI | TGCAGATTGAGTTGAGGCACT | GCAGTCGGCATTTCTGGAAC |
| AcSEP3 | GCCAGATGCTGCAGAGTAGT | TGCTCAATGGTCCCAAGTCC |
| AcAP3 | GCTCAAGAACACGCAAGTCC | TTTCGGAGTATTGCAGCCCC |
| Ac Actin | CTGGGATGACATGGAGAAGATT | GTTAAGTGGAGCCTCCGT |
| Acβ-tubulin | GTCTTCAGAGGCAAGATGAGCAC | TCAGTCCAGTAGGAGGAATGTCG |
